# Impact through research in education and studies in human society: A review of Australian Research Council ‘high-for-impact’ case studies

**DOI:** 10.1371/journal.pone.0302877

**Published:** 2024-05-31

**Authors:** Grace Jefferson, Rosita Henry, Marion Heyeres, Rhian Morgan, Louisa Tomas, Komla Tsey, Ines Zuchowski

**Affiliations:** 1 College of Arts, Society and Education, James Cook University, Douglas, Queensland, Australia; 2 Pathways, James Cook University, Douglas, Queensland, Australia; 3 The Cairns Institute, James Cook University, Douglas, Queensland, Australia; Universiti Pendidikan Sultan Idris, MALAYSIA

## Abstract

Research impact is an important measure of the effective transmission and ongoing contribution of research beyond the scope of initial research publication outputs; however, determining what constitutes ‘high-for-impact’ research can be difficult for specific fields of study. This review of the Australian Research Council’s *Engagement and Impact Assessment 2018* analyses high-for-impact case studies submitted in the fields of Education (n = 17) and Studies in Human Society (n = 11) with the aim of understanding and explicating how high impact research has been evidenced in these fields. The review was guided by three research questions that concern the identification of the key characteristics of high-for-impact case studies, their reported impacts, and the evidence researchers cite to support claims of impact. The review highlights an important limitation in how impact is defined and understood by researchers, particularly cultural and social impact. Half of the analysed case studies involved international engagement, with minimal partner collaboration in the global south and countries in the Indo-Pacific, despite the region’s strategic geo-political importance for Australia. Our findings draw into question the distribution of funding to universities and where investment might best be made for the highest potential return on research impact. Another key finding is that reported impacts across the domains of economy, society, culture, national security, public service, health, environment and quality of life offer little satisfactory evidence of impact, despite affording valuable insights into the nature of impact claimed. Accordingly, we conclude that to enhance the value of research and demonstrate impact in Education and Social Sciences, improved impact literacy is required among researchers. We assert that a better understanding of what constitutes impact and how it can be evidenced will support more impactful research designs. Wider adoption of the holistic anthropological definition of culture, which integrates values, practices and products, would enhance impact case studies by expanding their focus to include the broader cultural changes that underpin sustained social change. While the ARC engagement and impact agenda is a step in the right direction, improving the value of research for society will require a radical reconceptualisation of research and its funding, well beyond the current assessment framework. The Lowitja Institute’s research-for-impact framework [1] is proposed as an alternative approach to research priority-setting based on explicit evidence gap analysis.

## Introduction

Improving the value of research for society has become a major concern for policymakers globally over the past ten years. Evidence suggests that a high proportion of publicly funded research is wasteful due to a combination of factors, including limited translation of research evidence into policy and practice [2]. Not surprisingly, governments are no longer satisfied with researchers demonstrating only the quality of their research, but also require evidence of the impact and benefit of their research to society [3]. The goal is to motivate researchers and their institutions to improve the value of research, reduce ‘research waste’ [2], and demonstrate impact.

As part of its research engagement and impact agenda, the Australian Research Council (ARC) assesses research impact to facilitate knowledge translation, inform decision-making, demonstrate success and enable comparisons. The ARC defines research impact as “… the contribution that research makes to the economy, society, culture, national security, public policy or services, health, the environment, or quality of life, beyond contributions to academia” [4] (para7). The requirement that Australian researchers demonstrate the impact of their research is relatively new. An *Engagement and Impact Assessment* trial was held a decade ago [5], followed by an ARC pilot in 2017, which led to the introduction of the current *Engagement and Impact Assessment* scheme in 2018 [3].

Critical analyses of the usefulness or otherwise of current research impact assessment frameworks in helping researchers improve the value of their research and demonstrate impact are important. Without such analyses, there is a risk that national research impact case study assessment schemes, such as the ARC’s *Engagement and Impact Assessment* scheme [3], will become public relations or ‘box-ticking’ exercises [6]. While research can have positive impacts, it can also have unintended negative consequences [6], and to date, the research impact agenda in Australia assumes impact to be only positive. For Aboriginal and Torres Strait Islander peoples, for example, research ethics has always been about how to disrupt a history of exploitative and abusive research practices to ensure benefits and positive impacts [7]. As the Australian Government embarks on a review of national research policy, including the current ARC *Engagement and Impact Assessment* impact scheme [8], there is a great deal to learn from the efforts of Aboriginal and Torres Strait Islander organisations to improve the value of research for their communities.

The inaugural Australian *Engagement and Impact Assessment* scheme in 2018 involved research institutions developing and presenting case studies to evidence their research engagement and impact [3]. In this study, we reviewed and analysed ‘high-for-impact’–rated case studies, as identified in the 2018 ARC *Engagement and Impact Assessment* round. Our focus was to understand the characteristics of high-for-impact research of publicly available case studies in the Unit of Assessment (UoA) codes ‘*13 Education*’ and ‘*16 Studies in Human Society*’, the impacts claimed, and the evidence used to support them. This could inform the conceptualisation of future research projects, as identifying and defining impact in Social Sciences and Education can be traditionally challenging. Our objectives were to understand, through the lens of the ARC impact assessment framework, what impactful research looks like, and the value or otherwise of the framework in supporting researchers to improve the value of research, reduce waste and demonstrate impacts for society.

This paper begins by reviewing literature regarding the extent to which researchers are analysing research impact case studies submitted under impact assessment schemes in Australia, the United Kingdom and other countries, and the characteristics of these case studies. Next, aims and research questions are identified, and the research methodology is described, before the presentation of findings which explore the characteristics, evidence and reported impacts of the case studies. We conclude by drawing on an Aboriginal and Torres and Torres Strait research impact framework, the Lowitja Institute Research for Impact Tool [1, 6, 7] to argue that unless research funding agencies in Australia recognise and fund explicit research priority-setting, the current research engagement and impact agenda will do little to help researchers maximise research value and impacts.

## Literature review

It is important to identify and capture the impact of research to ensure that it is focused on and able to address complex social problems and improve outcomes for users [9]. Zardo [10] stresses that increasing research engagement without clear evidence of strategies that effectively lead to research uptake will not translate to research impact. While the move to presenting case studies for the assessment of research impact is an opportunity for researchers to maximise engagement and impact within their disciplines, the ARC *Engagement and Impact Assessment* presents conceptual and practical challenges for researchers. These include dealing with the significant time lag between conducting research and using resultant knowledge; identifying cause and effect; lack of funding to monitor impacts beyond the lives of projects; and the tight or limited deadlines within which researchers must write their case studies for submission [6]. A study by Dunlop [11], which analysed 166 politics and international relations case studies submitted by 56 universities as part of the UK Research Excellence Framework (REF) round in 2014, found that while generating an impact case study involves many people, the heavy burden of creating the narrative was often left to a single writer. Dunlop [11] asserts that the public often loses out as beneficiaries of research impact, due, in part, to the ‘top-down’ attitude applied to research design.

There are few examples of synthesised evidence of impact case studies that have been formally assessed, either in Australia or the UK. Only six studies were found across several disciplines, including the Arts, science, social science, political science, health, international studies and development, and engineering [5, 11–15]. The case studies assessed were from the UK REF 2014 round, with numbers ranging from 18–300 case studies per study [5, 11–15]. The three broad aims across the six studies identified included describing the characteristics of impacts, the applicability of the assessment process, and enablers and barriers of collaborative practices.

The studies reviewed applied varying lenses to the concept of research impact. For example, a study conducted in the UK by Robbins et al. [14] that investigated engineering and development research found that impact case studies commonly employed an “ecological modernization discourse” (p89) to shape economic, environmental. and social value claims. The authors reported that submissions regarding ‘high-tech innovations’ in the areas of industrial design and manufacturing tended to quantify economic impact while ameliorating environmental, health and safety challenges [14].

Wilkinson [15] examined 18 REF impact case studies submitted by one UK university in 2014, assessing the ways impact was generated and evidenced to gain an understanding of researchers’ perceptions of the benefits and challenges of evidencing impact, and the disciplinary variations in how research impact was conceived and demonstrated. They found the most common type of evidence for research impact were testimonials and supporting statements from individuals and organisations. Wilkinson [15] highlighted that even though most participants agreed on the importance of research generating impact, they had mixed views as to whether this has been influenced by the agendas of research councils and research assessment. However, evidencing impact was seen as a vehicle to raise the profile of individuals, groups and the university, and to encourage collaboration beyond the bounds of academia [15].

Brook [12] focussed on the end-user and what impact might look like in research concerning the Arts. Impact was measured in most case studies by the number of people who attended exhibitions, performances, or public art installations, or were otherwise reached. Data were collected about whether the reach was increased or extended, and what people did during and after the event. Brook [12] concluded that the evidence collected related to the quality or significance of the research, rather than its impact.

A report by the NCCPE [13] showed that collaborative approaches between museums and universities were most prominent in the areas of the Arts, Design, English and History. Dissemination was named the most frequently cited stage of the research cycle when contact occurred. Other data described the geographical reach of collaborations, the types and sizes of museums, or other partners involved [13].

Jones [5] reviewed the Australian Engagement and Impact Assessment (EIA) trial to assess the non-academic impact generated by a range of Australian universities and evaluated the EIA process. Findings highlighted that the quality of case studies during this preliminary phase could be improved. In addition to reach and significance, it was proposed that the contribution of research be considered as an assessment criterion [5].

This small sample of evidence highlights the potential value of publicly available research impact case studies as sources of data that researchers can use to understand the nature of reported impacts, if not the impacts themselves. Successful case studies serve as examples that researchers can use to tailor their methodologies towards more impactful research design. During the planning stages of research applications, researchers may also start thinking ahead about what kind of impact they are seeking to achieve [6, 16]; however, further examination of existing case studies is necessary to determine what the characteristics of impactful research might be and how to best evidence it.

The submitted ‘high for impact’–rated case studies in the 2018 ARC *Engagement and Impact Assessment* round amount to a substantial pool of data that can provide useful information to researchers about the enablers of impact [16], knowledge about the enablers of (and barriers to) case study assessment, and insights on how to maximise both engagement and impact within any given discipline. They offer an opportunity to explore how interpretations of the different domains of impact identified by the ARC, such as culture and society, influence impact claims and their assessment. Finally, the examination of the case studies that were assessed as impactful can also be used to reflect on the trends and limitations of the ARC *Engagement and Impact Assessment* framework.

## Methods

This review aimed to identify and analyse publicly available high-for-impact Education and Studies in Human Society research case studies from the 2018 ARC *Engagement and Impact Assessment* round, to highlight the challenges and opportunities for applied researchers looking to conceptualise and conduct impactful research in these fields. The fields of Education and Human Society were selected because of the authors’ disciplinary backgrounds, and because it is often more problematic to demonstrate impact in these areas, as opposed to areas with more quantifiable data, such as biomedical research.

A review and analysis of how impact was demonstrated in the case studies submitted for ARC assessment in the fields of Education and Human provides an opportunity to inform research practice not only in these two fields, but also in other research fields. Reviewing case studies that were assessed as ‘high-for impact’ enables identification and critical analysis of how impact was defined, the criteria used to assess research as impactful, and the criteria that may have been missing from consideration. High-for-impact case studies in any field are not representative of the full range of impactful research that is conducted in Australia. The case studies submitted for assessment specifically relate to what is sometimes categorised as strategic and/or applied research, as opposed to ‘pure research’ or ‘blue-sky research’, which may also be impactful, depending upon how impact is defined and the time frame of the impact.

Institutions submitted their impact study under their chosen UoA category. The UoA is defined as a two-digit FoR code that has three levels. In this paper, we use the term UoA for the first level of categorisation, *Education* (UoA-13) and *Studies in Human Society* (UoA-16). Education includes four research groups: *1301 Education Systems*, *1302 Curriculum and Pedagogy*, *1303 Specialist Studies in Education* and *1399 Other Education* [17]. Studies in Human Society include nine research groups: *1601 Anthropology*, *1602 Criminology*, *1603 Demography*, *1604 Human Geography*, *1605 Policy and Administration*, *1606 Political Science*, *1607 Social Work*, *1608 Sociology* and *1699 Other Studies in Human Society* [18].

This review of ‘high for impact’–rated case studies corresponds to the three elements of the ARC impact assessment template’s key features of the case studies, reported impacts, and evidence supporting impact claims. Thus, it was guided by the following research questions (RQ):

RQ1 What are the key characteristics of identified high-for-impact case studies?
RQ2 What are the reported impacts of the case studies?
RQ3 What evidence do researchers cite to support their claims of research impact?

The studies analysed were publicly available and published on the ‘*Engagement and Impact–Impact Studies*’ section of the ‘*ARC Data Portal*’ website [19]. The inclusion criteria were case studies that reported either under the codes *13 Education* or *16 Studies in Human Society* as their UoA. Twenty-eight relevant studies were identified (Tables 1 and 2). For the purposes of this review, each case study was coded for ease of reference (Education case studies: ECS1, ECS2, etc., or Studies in Human Society case studies: HSC1, HSC2, etc.). The data that were analysed comprised information available on the individual ARC webpages for each study; that is; an overview of the research (e.g., institution, socio-economic objectives, Australian and New Zealand Standard Industrial Classification and keywords), a brief 1-paragraph summary of the impact, and the details of the impact (approximately 1–2 pages of text).

**Table 1:** A summary of the high-for-impact Education case studies (n = 17).

Short title and link to case study (code)	Lead Institution	Summary of Reported Impact
Equity And Fairness According to Student Need (ECS1)	Victoria University	Improved funding models that are designed to be more equitable, accountable, and efficient and distributing funding more fairly and transparently, used in five Australian states and territories, with independent reviews evidencing greater flexibility and autonomy in addressing student need.
Using Children’s Voices to Build Better Worlds (ECS2)	University of South Australia	Empowerment of children to be active contributors to decision-making resulted in improved policies and practices that impacted 419,250 South Australian children/young people’s participation, learning and wellbeing. The research’s principles were embedded into UNICEF’s Child-Friendly Cities Toolkit, with 30 Australian communities seeking Child-Friendly accreditation. Improved preschool literacy in Fiji, impacting 273 families and developing resources for around 40,000 pre-schoolers.
Collaborative Pedagogies in Low Socio-Economic Status (SES) Communities (ECS3)	Western Sydney University	Improved classroom teaching practices in preschools and schools in poor, disadvantaged communities in Australia and Chile. Teachers improved their research practice and increased parental involvement in children’s learning. Dramatically improved performance on national literacy and mathematics tests in Chilean children. Increased motivation, confidence and learning engagement. Development of new national standards for early childhood teacher education in Chile. Strong influence on school education policy and teacher professional development in Australia.
Mathematics Education in Pre- and Primary School Contexts (ESC4)	Macquarie University	Changes in professional mathematics teaching practice and family involvement in children’s education resulted in significant mathematical learning gains for pre- and primary school children. Curriculum developers, teachers, educators, pre-service teacher education students, families and children have benefited from the mathematics teaching programs and assessment tools developed at Macquarie.
Developing and Testing Indicators for Inclusive Education (ECS5)	Monash University	Enhanced teacher capabilities in teaching students with diverse learning needs in Australia, The Pacific Islands and Bangladesh. Development of two resource tools to determine the level of adjustment needed for students with disabilities, and to personalise learning for those students. Policy impact in Australia and overseas regarding the inclusion of learners with disabilities into mainstream classrooms.
Integrate Numeracy Learning across the Curriculum (ECS6)	The University of Queensland	Changes in the pedagogy underpinning the Australian Curriculum turned the teaching of mathematics towards solving problems in other scenarios. Local, national, and international impact on mathematics curriculum and pedagogy.
The Students with Additional Needs Program (ECS7)	The University of Melbourne	Developed an integrated program of curriculum, teaching, assessment, and reporting resources that provide schools with access to high-quality, research-based materials to support their professional practice. Improved assessment and reporting of Australian students with intellectual disability or developmental delay. The research resulted in the widespread use of system-level resources and the program is recognised as a government-endorsed professional learning option as part of mandatory teacher registration in Victoria.
Representation Inquiry in Science Teaching and Learning (ECS8)	Deakin University	Significant contribution to the reformed science education both in Australia (particularly in Victoria) and internationally. The Representation Construction Approach involves students taking an active role in making, negotiating, refining, and justifying their own representations in a guided inquiry process. Aligned with the knowledge-building practices of scientists, the Representation Construction Approach impacted policy and practice and positively changed the way teachers and students think about and engage with science.
School Bullying, Violence and Wellbeing in Schools (ECS9)	The Flinders University of South Australia	The anti-bullying resource PEACE Pack has significantly increased policy- and program-level awareness of peer bullying in schools and the consequent reduction of wellbeing in children. The PEACE Packs have contributed to the reduction of peer bullying and the improvement of learning environments and outcomes in schools locally, nationally, and internationally.
Needs of at-risk learners in Numeracy and Literacy (ECS10)	The University of New England	Participants of the individually focused, 30-week program Quicksmart consistently achieved skill development equivalent to that normally achieved over 2–3 years of schooling for a peer, as well as increased self-confidence and self-esteem.
Simulation Innovation in Teaching and Learning (ECS11)	Central Queensland University	Patents have been secured and international licenses issued to providers in the UK and USA. In-service training has been provided to clinicians at over 25 hospitals in Australia, Nepal, Singapore, UK, and the USA. Domestically the technique is being used as a training aid to address the National Patient Safety Standards.
Digital Technologies in Secondary Schools (ECS12)	Edith Cowan University	The application of the research has created new contexts for learning through effective use of Information and Communication Technology–bringing demonstrable immediate benefits and longer-term impact for national and state education authorities, school systems and sectors, schools, teachers, and students. Within the reference period, Edith Cowan University’s research has had wide-reaching impact, influencing practice in schools, practice across systems and sectors, education policy development, and implementation associated with curriculum, pedagogy, and assessment at state and national levels.
The Quality Teaching Model and Quality Teaching Rounds (ECS13)	The University of Newcastle	The Quality Teaching model of pedagogy and Quality Teaching Rounds approach to teacher development stand out for their demonstrated impact not only on teachers, but on schools and school cultures, school systems and, ultimately, school students. Quality Teaching and Quality Teaching Rounds are embedded in education systems and education policy, especially in New South Wales and the Australian Capital Territory. Through published outputs and strong engagement with end-users, this research has profoundly shaped how quality teaching is conceptualised, taught (to preservice and in-service teachers) and researched in Australia.
Childhood Environmental Education and Sustainability (ECS14)	Southern Cross University	Development of a preschool program that integrated learning about sustainability and healthy eating through different types of play. Increased children’s environmental knowledge and shifting environmental behaviours. Involvement of children and young people in the writing of teaching programs for schools, developing art exhibitions, networking about environmental matters online and taking part in a Climate Change Challenge. Published books and articles, seminars about sustainability teaching programs for teachers and trainee teachers around the world.
Resolving Challenges in Early Childhood Learning (ECS15)	Australian Catholic University	Development of a framework that has been disseminated across the early childhood sector nationally, as the central design concept in the Early Language Learning Australia apps. The use of the apps supports attainment of the Early Years Learning Framework (2009) outcomes 1, 2, 4 & 5 relating to children’s sense of identity, connection, confidence, and effective communication. The Framework has been adapted to other knowledge areas such as STEM education and recommended by a UNESCO report to support sustainability skills learning internationally.
Digital Technologies in School Education (ECS16)	University of Technology Sydney	Increased and improved use of digital technologies in school education in Australia and overseas. Transformed teachers’ understanding of and competence in integrating these technologies into their classroom practice, increased schools’ uptake and investment in them, and directly enhanced students’ creative and critical thinking skills and engagement with STEM and other subjects. Teachers benefitted also from new curriculum materials, tools and resources arising from the research. New pedagogical frameworks produced by the research had an impact on government policymaking in Australia and Scotland, and informed Microsoft’s thinking about educational applications of digital technologies.
Shaping National and International Educational Measurement Approaches and Practices (ECS17)	The University of Western Australia	Development of an innovative assessment method to enable schools to streamline school assessment processes across the nation. Brightpath has been adopted by approximately 500 schools and used for 300,000 assessments of Australian students. The research has impacted Australian education communities through critical involvement with the National Assessment Programme—Literacy and Numeracy involving over 1 million student participants per year; and the high-stakes Western Australian Certificate of Education affecting 26,000 high school students each year.

**Table 2:** A summary of the high-for-impact Studies in Human Society case studies (n = 11).

Short title and link to case study	Lead Institution	Summary of Reported Impact
Police Engagement with the Public (HCS1)	The University of Queensland	Development of a world-first structured dialogue, changing how police interact with people from different backgrounds. This dialogue has empowered police in Australia and overseas to influence greater mutual dignity and respect during encounters by helping citizens better understand the reasons for police actions. The subsequent improved perceptions of police as trustworthy and legitimate improved some driver behaviours in Australia and how police engage with citizens in this country, the United States, England, Scotland, and Turkey.
Australia’s International Traders’ Competitiveness (HCS2)	Charles Sturt University	Key catalyst to the introduction of the Government’s Australian Trusted Trader program in 2016. Australian businesses are provided with international trade facilitation benefits, such as reduced regulatory requirements both in Australia and in certain export markets that increase international competitiveness. In support of the Government’s decision to enter into international Mutual Recognition Agreements with key trading partners. To date some 160 Australian companies have joined the Australian Trusted Trader program and are reaping the commercial benefits.
Practical Tools for Creating Sustainable Cities (HCS3)	Western Sydney University	Development of a holistic framework that gives equal emphasis to the cultural and political domains, to assess the sustainability of cities. The method was adopted by cities in Australia, Europe, Africa, and the Americas, as well as by international organisations such as Metropolis. It transformed how they evaluated urban sustainability and guided new projects to address sustainability challenges. The method was also used by non-government organisations to develop policy in areas such as climate change adaptation and sustainable food systems.
Reducing the Road Toll with a Young Person-Centred Workshop (HCS4)	RMIT University	Development of an innovative approach to road safety education that focuses on young people’s capabilities and so empowers them to engage in safer road use behaviours (Fit to Drive). Demonstrably making young people safer road users, the approach has come to form the heart of road safety education programs delivered across Australia.
Understanding and Adapting to Experiences of Loss and Grief in Children, Young People and Adults (HCS5)	Southern Cross University	Development of the Seasons for Growth (SfG) program to support children and young people following death, separation, divorce, and other loss experiences. At the request of other groups, SfG was adapted to support refugee children, young people in suicide ‘hotspots’ and children involved in natural disasters. The program was also adapted for adults experiencing loss, Indigenous people, prisoners, and parents of children in the program. Since 1996, 260,000 children, young people, and adults in 5 countries have taken part in SfG and 82,000 were involved between 2011–16.
The Fight for the Right to Work (HCS6)	Curtin University of Technology	Significant contribution to the changes in policy that granted asylum seekers living in the community the right to work while they awaited the outcome of their claims.
Criminological and Sociological Impacts of Fly in Fly Out (FIFO) Work (HCS7)	Queensland University of Technology	Considerable impact on end users–including Fly-in and Fly-out (FIFO) workers, and residents of rural and regional communities. Influenced the establishment of a national Australian Inquiry into the Impact of FIFO on Rural and Regional Australia. This has led to new laws in Queensland banning mining companies from using a 100 per cent FIFO workforce.
Reforming Child Protection Policy and Practice (HCS8)	University of South Australia	The University of South Australia has shaped national intervention frameworks: National Framework for Protecting Australia’s Children with 70 national actions, and the Royal Commission into Institutional Responses to Child Sexual Abuse; identifying priority action areas and developing indicators for monitoring progress.
Tackling Rural Crime – Better Reporting and Prevention Strategies (HCS9)	The University of New England	The research contributed to police operations in New South Wales and Queensland, informed training of police recruits and legislation relating to rural crimes, as well as assisted in raising community awareness and simplifying citizen reporting of rural crimes.
Using Social Research to Improve Government Policy and Regulatory Decision-Making (HCS10)	University of Technology Sydney	End-user research participation to include consumer views in the decision-making process which led to Sydney Water’s decision to recommend a reduction in the price of water. The New South Wales Independent Pricing & Regulatory Tribunal accepted the recommendation, delivering savings of $720M over 4 years to 1.8M households and businesses across Sydney and the Illawarra. The research also enabled government agencies dominated by economics and engineering to develop new capabilities for engaging with the citizens they serve.
Enhancing Policy-Making and Public Dialogue on the Future of Cities (HCS11)	The University of Western Australia	Inter-sectoral partnership that focused on major urban policy concerns related to globalization, economic development, demographic change, urban liveability, and social equality stimulated debate across business, community, government, and individuals about the future of cities. The partnership draws on an active and ongoing strategy of engagement with stakeholders, and actively seeks to inform policy by improving the quality of evidence used by urban decision-makers.

Using an exploratory mixed methods approach, qualitative and quantitative data were extracted from the relevant case studies in accordance with pre-defined criteria and analysed in two phases. In Phase 1, a descriptive analysis of the case studies was completed to identify and quantify key characteristics. In Phase 2, a qualitative ‘document’ analysis was performed to identify themes within the reported impacts of the case studies.

### Phase 1: Characteristics of the case studies

This phase of data analysis concerned the characteristics of the case studies (RQ1). In this phase, two authors worked as a team to analyse the Education case studies (n = 17) and two other authors worked on the Studies in Human Society case studies (n = 11). Qualitative data included information about the reported impact, approach to impact (partnerships, institutional support and other factors that enabled the impact), beneficiaries of the outcomes of the research, research locations, the country where the impact occurred, and associated research output types. This qualitative data was collated within each study area and represented as infographics to provide a visual comparison of the various approaches to impact and beneficiaries as revealed in the studies. The studies, including each type of evidence for each area of study (Education and Studies in Human Society), were compared using visual representations.

Quantitative data comprised information about the UoA, the institution that submitted the case study, the study focus, and whether the case study included Aboriginal and Torres Strait Islander research. Other quantitative data examined included the number of research outputs, numbers of research collaborator nations/research partners and/or users involved, number of years since publication/research outputs to reported impact, and the number of regional and ‘Group of Eight’ (Go8) university research leaders/partners involved in the studies (*The Group of Eight* comprises Australia’s leading research-intensive universities). Data were analysed to find measures of centre and spread for comparison between the study areas, and compared through histograms, tables, and other visual representations.

### Phase 2: Research impact and evidence

A document analysis [20] was employed to identify and analyse reported research impacts and how they were evidenced (RQ2 and RQ3). In this phase, the authors worked in small teams to analyse the Education case studies (n = 17) and the Studies in Human Society case studies (n = 11). The authors subdivided the case studies and analysed them individually after jointly analysing a sample of three and agreeing on potential themes. This involved an inductive thematic approach [21]; carefully reading and re-reading the case studies and identifying segments of text that identified impacts and how they were evidenced. The impacts were organised into categories under domains that aligned with the ARC definition of impact: economy, society, culture, national security, public policy or services, health, the environment, and quality of life. The text segments were initially colour coded to identify ‘like impacts’, which yielded a range of ‘categories of impact’ (i.e., themes). The impact categories and the text segments from which they emerged were discussed and refined between the authors in each team until consensus was reached. This process of refinement included a discussion of how societal and cultural impacts were represented in case studies and an agreement to apply anthropological understandings of society and culture to the analysis. Finally, all authors reconvened to share and discuss their collective findings, and further refined the categories of impact through a series of meetings. During this process, careful consideration was given to objectivity and sensitivity, ensuring the categories of impact were represented fairly [21].

## Findings

### Phase 1: Characteristics of the case studies

The numerical analysis of the high-for-impact case studies highlights key findings concerning research institution type and location, countries affected and involved in the research across the selected period, numbers and types of research partners and beneficiaries, and outputs. Several trends were found in research group composition and primary locations, the communities impacted by the research, and the characteristics of the cited evidence of impact.

#### Research institutions

Comparisons of research contributions from regional and metropolitan universities found that less than half were from regional institutions, seven of the seventeen Education case studies and four of the eleven of the Human Society case studies. Only four of the Education case studies and two of the Human Society case studies were authored by Australia’s Go8 member universities.

#### Research focused on and led by Aboriginal and Torres Strait Islander people

Aboriginal and Torres Strait Islander-focused and Indigenous-led research has been identified as a key focus for the Australian Government [22]. In both the Education and Human Society case studies, only three of the studies, had an Aboriginal and Torres Strait Islander focus or beneficiaries.

#### Research collaboration

**Fig 1:**
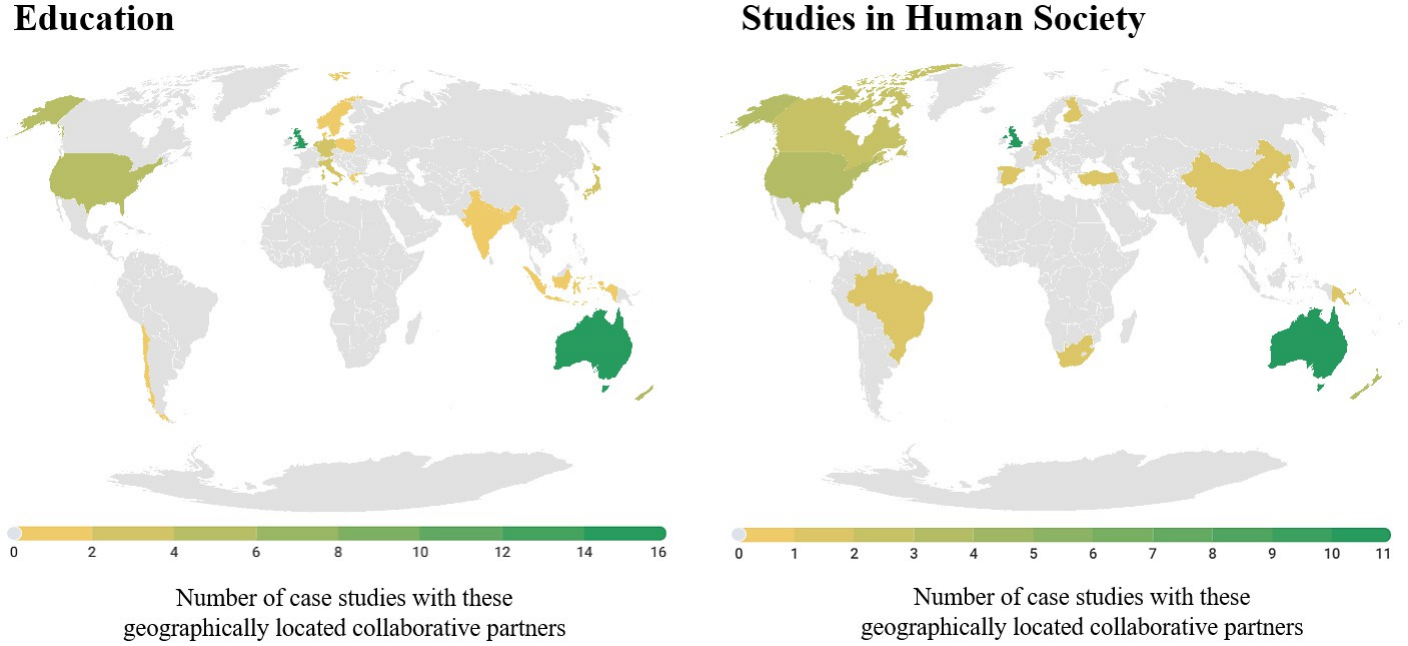
Geographical distribution of collaborative partners for research in Education and Human Society high-for-impact case studies.

International collaborators were found to be located all over the world (Fig 1). There was a noticeable difference, however, between the geographic location of collaborators in Education case studies compared to Human Society case studies, with the latter demonstrating a broader range of partner nations and a focus on collaborations across America, Canada, and China. Education case study partner nations were mostly represented by the UK, the United States (US), and New Zealand.

**Fig 2:**
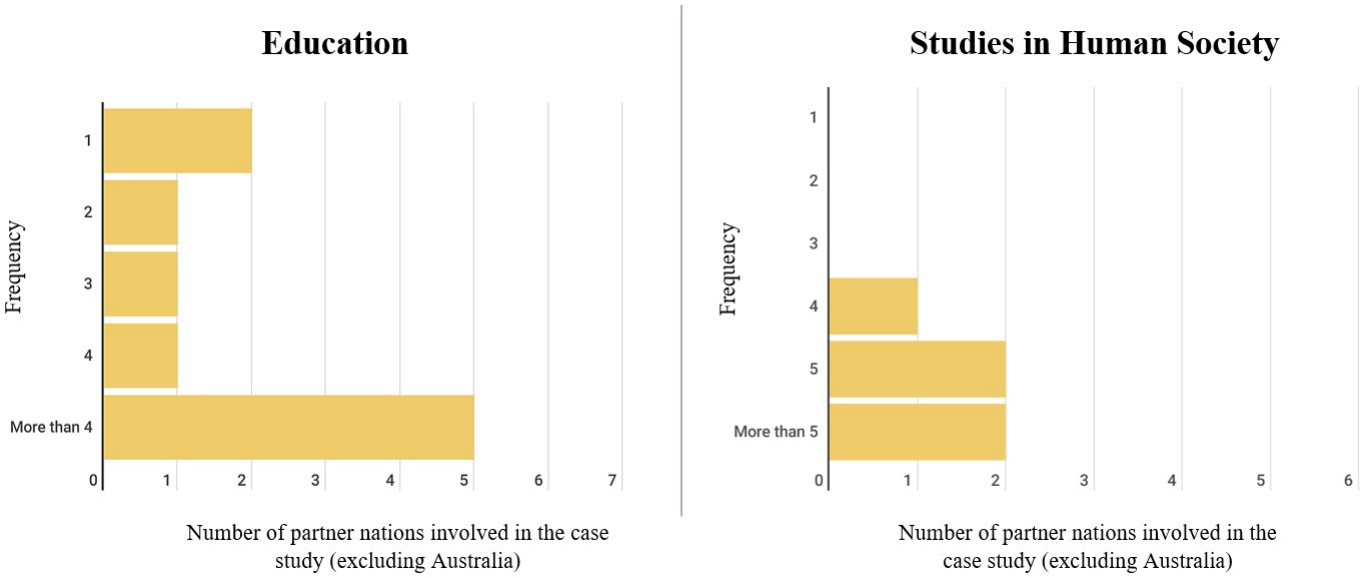
Numbers of international collaboration partner nations for research in Education and Human Society high-for-impact case studies.

Collaboration featured in approximately half of the research in each field of study; that is, ten of the seventeen Education case studies and six of the eleven Human Society case studies. The most common number of international collaboration partners is five or more nations in both Education and Studies in Human Society (Fig 2).

#### United Nations economic status of countries impacted by the research

The data for research partners were analysed by categorisation of nations as *developing*, *transitioning*, *or developed* in accordance with the United Nations Country Classification [23]. Most research partners were from developed countries as evidenced in nine of the Human Society case studies and eleven of the Education case studies. The remaining case studies were from developing countries, and none were from transitioning nations.

#### Evidence supporting impact

**Fig 3:**
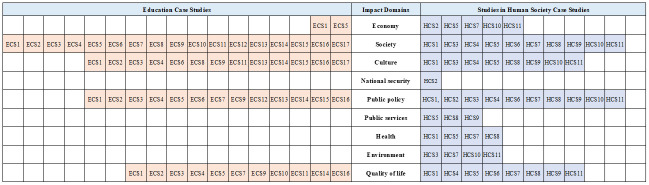
Evidence supporting impact claims for Education and Human Society case studies by identified impact domain.

Broad categories of evidence supporting impact claims in each study are identified in Fig 3. Education case studies were more likely to include evidence of impact related to economy and society, whereas Human Society studies have a broader range of impact domains identified from the provided evidence, with national security, public services, health and environment domains identified for Studies of Human Society only.

#### Research outputs

**Fig 4:**
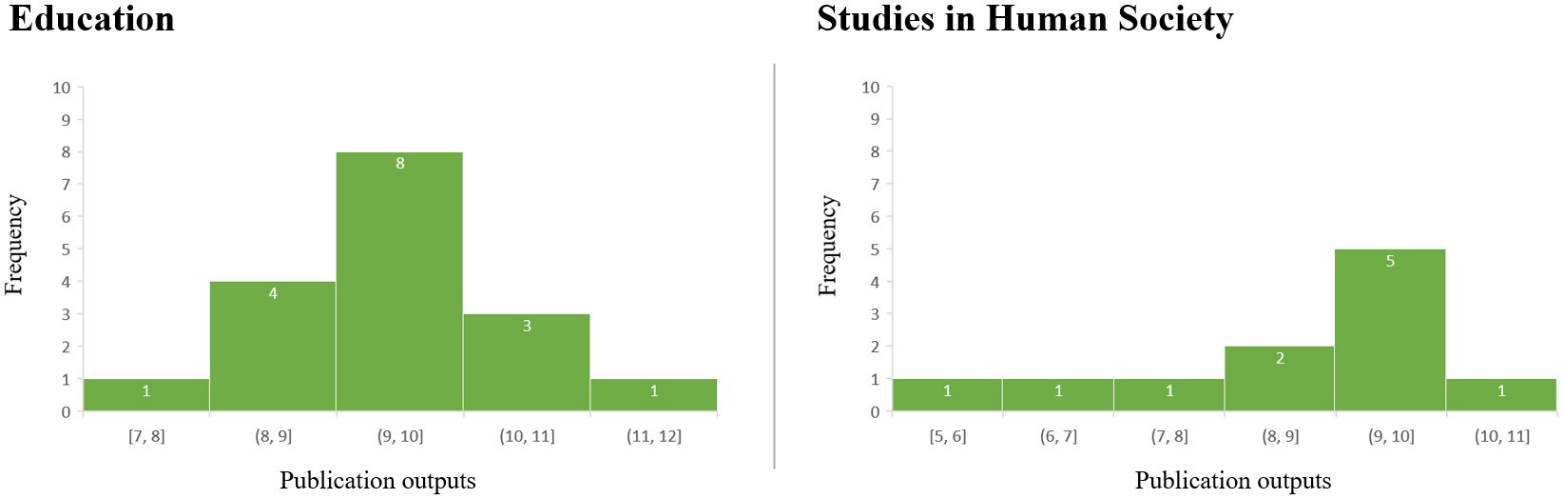
Number of research outputs in Education and Human Society high-for-impact case studies.

Research outputs included book chapters, reports, the development of frameworks and implementation of policy, as well as other outputs both locally and internationally. These results were consistent with a mean of 9.9 and 9.0 outputs per study for Education and Human Society case studies, respectively, and a median and mode of 10.0 associated outputs for both UoA (Fig 4).

#### Timing of reported research impacts

**Fig 5:**
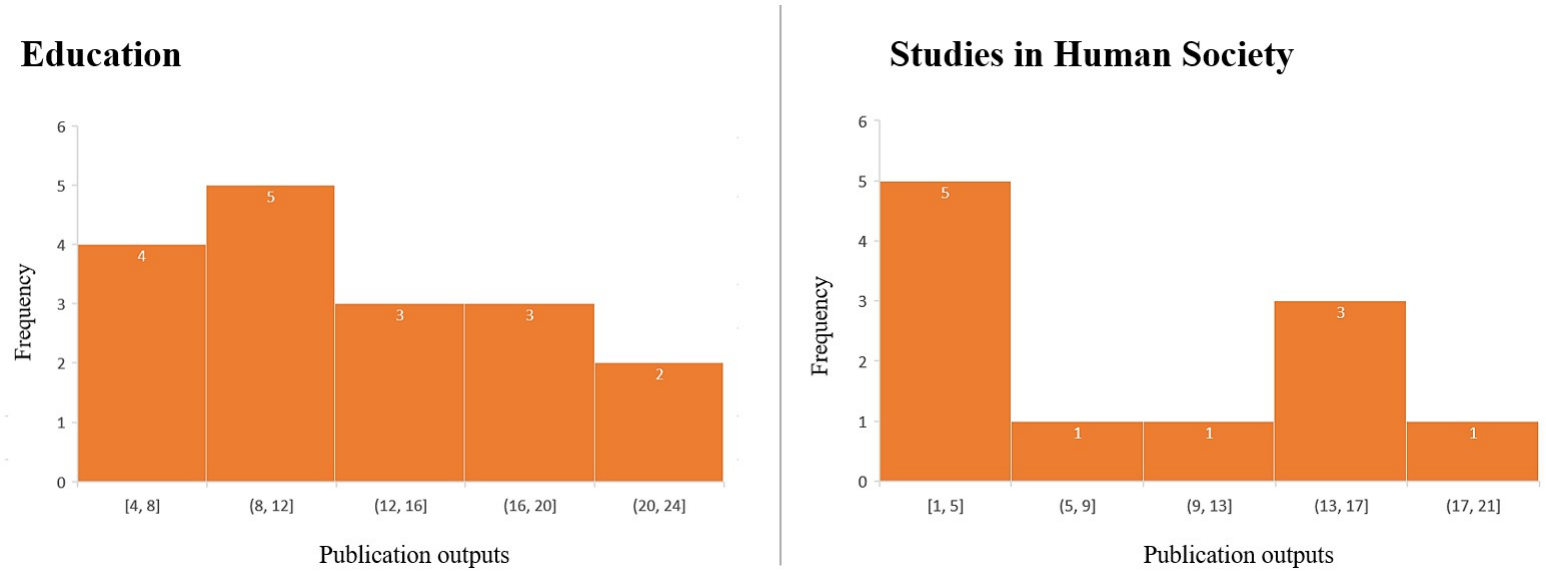
Statistics for the number of years between the publication of research findings and submission of the research high-for-impact case studies in 2018 in Education and Studies in Human Society.

The number of years between the publication of research findings and submission of the research impact case studies in 2018 is shown in Table 3 and Fig 5. Note that the maximum value in Table 3 of ‘20 years’ between publication and submission for impact recorded for Studies in Human Society was used for evidence with an interval given of ‘20+ years’ (the evidence was produced more than 20 years ago but with no specific timeframe), so the lower bound of 20 was used for this data point. Similarly, the data point of ‘15+ years’ was entered as 15 years for Human Society studies.

**Table 3:** Statistics for the number of years between the publication of research findings and submission of the research high-for-impact case studies in Education and Studies in Human Society.

Field	Mean	Median	Mode	Min	Max
Education	13.2	12	7	1	20
Studies of Human Societies	8.7	9	2	4	20

Over half of the identified research outputs in each area of study were published within 12 years, with nine of the Education and seven of the Studies in Human Society high-for-impact case studies. The median number of years between the publication of research findings and submission of the research impact case studies is 12 years for Education and nine years for Human Society case studies; however, the mode is lower (7 years and 2 years, for each area, respectively).

### Phase 2: Research impact and evidence

The findings arising from the qualitative document analysis of the Education and Studies in Human Society high-for-impact case studies are presented below.

### Education case studies

Table 4 provides a summary of the thirteen impact categories identified in the Education Case Studies. The impacts claimed in these case studies most frequently were improvement in education policies and practices (n = 13); the development of educational resources and infrastructure (n = 13); teacher professional development and training (n = 13); improved participation and engagement of key stakeholders (n = 12); and the development of new models and frameworks for education, including curricula (n = 11).

**Table 4:** Summary of the impact categories that emerged from the Education case studies.

*Impact Domains*	*Categories of Impacts*	*FoR 13 Case studies*	*n*
Economy	Improved education funding models	ECS1 ECS5	2
Society	Development of educational resources and infrastructure	ECS1 ECS2 ECS4 ECS5 ECS7 ECS8 ECS9 ECS11 ECS12 ECS13 ECS14 ECS15 ECS16	13
	Teacher professional development and training, and workforce capacity	ECS2 ECS3 ECS4 ECS5 ECS6 ECS7 ECS8 ECS9 ECS10 ECS11 ECS15 ECS16 ECS17	13
	Improved stakeholder participation and engagement	ECS1 ECS2 ECS3 ECS4 ECS8 ECS10 ECS12 ECS13 ECS14 ECS15 ECS16	11
	Improved teaching practices and pedagogies	ECS3 ECS4 ECS7 ECS2 ECS10 ECS12 ECS13 ECS15 ECS16	9
	Improved student learning outcomes	ECS2 ECS3 ECS4 ECS8 ECS1 ECS12 ECS15 ECS16	8
	Impacts on school-based assessment and reporting	ECS4 ECS5 ECS7 ECS8 ECS9 ECS12 ECS17	7
Culture	Development of new models and frameworks (including curricula)	ECS1 ECS3 ECS4 ECS6 ECS8 ECS9 ECS13 ECS14 ECS15 ECS16 ECS17	11
	Cultural impacts	ECS2 ECS4 ECS11 ECS14 ECS15 ECS16	6
	Impacts on advancing further research and inquiry	ECS1 ECS6 ECS8 ECS15 ECS16 ECS17	6
Public policy	Improved education policies and practices	ECS1 ECS2 ECS3 ECS4 ECS5 ECS6 ECS7 ECS9 ECS12 ECS13 ECS14 ECS15 ECS16	13
Quality of life	Improved social outcomes, including well-being	ECS2 ECS3 ECS5 ECS7 ECS9, ECS10 ECS11 ECS14 ECS16	9
	Impacting disadvantaged communities	ECS1 ECS3 ECS4 ECS10 ECS16	5

#### Impact reach

Sixteen of the seventeen case studies reported impacts in Australia at a community, state, or national level. Impacts that had national reach included those that shaped national research agendas (ECS14); or informed the development of national curricula, standards, or policies (ECS1, ECS8, ECS10, ECS11, ECS12). Fourteen studies reported international reach including uptake of professional development seminars in Poland and the United States (ECS14) and changes to international student assessment (ECS8).

#### Public policy—Improved education policies and practices

Improved education policies and practices at community, state, national and international levels were reported in fourteen of the ECS. For example, impacts included public policy reform in relation to children’s rights, parent engagement and teacher education standards for Early Childhood Education (ECS3), educational policy development and decision-making concerning the adoption and application of new Information & Communication Technologies in schools in Western Australia (ECS12); and impacts on the OECD’s approach to international numeracy assessment (ECS 6).

#### Society—Development of educational resources and infrastructure

The research impact reported by thirteen ECS concerned the development of educational resources and infrastructure. Curriculum resources were most frequently reported, such as educational websites and online learning platforms (ECS5, ECS7), a science textbook for preservice teachers (ECS8), curriculum resource packages (ECS13), and the design and construction of outdoor play spaces and equipment (ECS2).

#### Society—Teacher professional development and training, and workforce capacity

Thirteen case studies reported on teacher professional development and training. These impacts chiefly concerned the development of professional development resources, and packages and frameworks for in-service and preservice teachers on topics such as collaborative pedagogies (ECS3), inclusive education (ECS5), mathematics and numeracy education (CS4, ECS6), and science education (ECS8) and training (ECS2, ECS4).

#### Society—Improved stakeholder participation and engagement

Eleven case studies reported improved stakeholder participation and engagement in education. For example, engaged children’s voices in community decision-making (ECS2), increased engagement of parents and communities (ECS4), engaged and interested secondary students (ECS16).

#### Culture—Development of new models and frameworks (including curricula)

Eleven case studies reported research impact by way of new models and frameworks for education, including new curricula. For example, ECS3 reported on the development of a pedagogical framework aimed at improving outcomes for students in disadvantaged schools. Several studies reported impacting the development of curricula, such as the Australian Curriculum (e.g., in learning areas such as mathematics [ECS4] and science [ECS8], and the General Capabilities, such as Numeracy [ECS6]), and the Early Years Learning Framework (ECS4).

### Studies in Human Society case studies

A summary of the fourteen impact categories that emerged from the Studies in Human Society case studies is presented in Table 5. The most frequently reported impacts concerned changes in public policy (n = 8), changes in understanding (by way of new frameworks/methods/analysis, n = 8), changes in organisational practice (n = 6), improved community engagement (n = 6), improved trade and business (n = 5), and improved social justice or community well-being (n = 5). All studies cited impacts across at least two different impact domains.

**Table 5:** A summary of the impact categories that emerged from the Human Societies case studies.

*Impact Domains*	*Categories of Impact*	*FoR 16 Case Studies*	*n*
Economy	Improved trade and business	HCS2 HCS5 HCS7 HCS10 HCS11	5
Society	Change in organizational practice (business/org/public sector)	HCS1 HCS3 HCS8 HCS9 HCS10 HCS11	6
	Improved Community engagement (including in government decision-making)	HCS4 HCS6 HCS7 HCS9 HCS10 HCS11	6
	Change in individual behaviour (private citizens/general/public/specific demographics)	HCS1 HCS4 HCS5 HCS9	4
Culture	Change in (or development of or improved) understanding (new frameworks/methods/analysis)	HCS1 HCS3 HCS4 HCS5 HCS8 HCS9 HCS10 HCS11	8
National security	Improved Law & order (policing & public safety, crime prevention)	HCS2	1
Public policy	Change in (or development of) policy (economic, social, environmental, cultural)	HCS1, HCS2 HCS3 HCS4 HCS6 HCS7 HCS8 HCS10 HCS11	9
	Change in (or development of) Law/Legislation	HCS1 HCS7 HCS8 HCS9	4
Public services	Change in (or development of) public services	HCS5 HCS8 HCS9	3
Health	Improved health outcomes	HCS1 HCS5 HCS7 HCS8	4
Environment	Improved environmental or ecological conditions	HCS3 HCS11	2
	Improved Natural Resource Management	HCS3 HCS7 HCS10 HCS11	4
Quality of life	Improved Social justice/ Community wellbeing	HCS1 HCS6 HCS7 HCS9 HCS11	5
	Improved wellbeing of children/youth	HCS4 HCS5 HCS8	3

#### Public policy—Change in public policy and/or legal frameworks

Nine of the eleven case studies highlighted impacts on government policy or practice as a key research outcome. Policy impacts were identified at local (HCS3, HCS10, HCS11), state (HCS1, HCS4, HCS7, HCS10, HCS11), federal (HCS2, HCS6, HCS8) and international levels (HCS3), with two case studies reporting impacts at multiple levels of government (HCS10, HCS11). Legislative changes were reported in the areas of law enforcement (HCS1, HSC9), workers’ rights (HCS7), and child safety (HSC8). Impacts on governmental strategic initiatives included road safety (HCS4), rural crime (HCS9), child protection (HCS8), sustainable urban planning (HCS10, HCS11) and resource management (HCS10).

#### Culture—Change in understanding (new frameworks/methods/ analysis)

Research that involved the development of new analytic frameworks, methods or training approaches was reported in eight of the eleven studies. While not all these studies mentioned the word culture, we have listed them against cultural impact as they claimed impact on collective ideas or ways of understanding, in accordance with the definition of culture we have adopted in the current study; that is, the shared values, understandings and relations that underpin society [24, 25]. Five of these studies developed training programs (HCS1, HCS3, HCS4, HCS5, HCS9), two were focused on law enforcement (HCS1, HCS9), one on road safety (HCS4), one on urban planning (HCS3), and one on grief and loss (HCS5). The development of a new method for understanding a problem was also reported in the case study focused on child safety (HCS8). Two case studies reported the development and implementation of new analytic methods, specifically in the areas of urban planning/sustainability (HCS3) and resource management (HCS10). HCS10 also reported a change in organisational culture.

#### Society—Change in organisational practice

Six case studies reported improvements to public sector business practices or strategies as a key impact. Three case studies helped implement new strategic approaches to decision-making or planning (HCS3, HCS10, HCS11) and two implemented new organisational structures to impact practice (HCS7, HCS9). The methods used to improve business practices were quite diverse. For example, HCS1 created a new dialogic method for public engagement in law enforcement and others integrated new stakeholder groups into consultation processes in resource management (HCS8, HCS11).

#### Society—Improved community engagement

Improvements to community engagement or public discourse were cited as areas of impact in six case studies (HCS4, HCS6, HCS7, HCS8, HCS10, HCS11). Studies reported raising awareness of specific social issues using multiple engagement methods; for example, news media (HCS4, HCS6, HCS7), the publication of reports or resources (HCS4, HCS7, HCS9, HCS11) and community/public forums (HCS6, HCS7, HCS11). One case study reported raising awareness of issues surrounding rural crime via a community training program (HCS9), while two studies reported increased community engagement in public sector decision-making in resource management (HCS10) and road safety (HCS4).

#### Economy—Improved trade and business

Five case studies claimed to have had economic impacts. For example, HCS2 reported that the research improved trade and business by enhancing the competitiveness of Australia’s international traders. HCS7 reported that the research was used in the assessment of economic impacts relating to ’fly in-fly out’ workers in Queensland.

#### Quality of life—Improved social justice/community well-being

None of the eleven studies use the phrase ‘quality of life’ or specifically claimed to have contributed to enhanced quality of life. However, enhanced quality of life may be assumed to have been the target for most, if not all, of the studies. In particular, at least eight of the case studies could be understood as enhancing the quality of life through improving social justice. Three of these were focused on children or youth wellbeing (HCS4, HCS5, HCS8), while five were focused on community wellbeing more broadly (HCS1, HCS6, HCS7, HCS9, HCS11).

## Discussion

The ARC Engagement and Impact agenda presents opportunities for researchers to demonstrate the societal benefits of their research. Currently, researchers are not funded to track impact beyond the immediate scope of their studies; yet they are required to write and submit impact case studies within tight assessment timeframes. This makes meaningful development of an evidence-base for research impact difficult. As researchers, we need to be more proactive in our approaches. The publicly available ARC case studies are valuable sources of data to understand the enablers and characteristics of high-for-impact research. Our analysis of case studies in Education and Studies in Human Society has revealed several significant factors that might foster engagement and impact of research in these two fields. We first discuss these factors with a view to improving impact literacy among researchers and assisting researchers in the planning and design of research. We then draw on insights from the Lowitja Institute research impact framework [1, 6] to argue why a radical reconceptualisation of research well beyond the current ARC *Engagement and Impact* agenda is required to support Australian researchers to improve the value of their research, reduce waste, and demonstrate impact.

### Characteristics of the case studies

The broad range of characteristics identified included depth and breadth of research collaborations, research aims and reported impact. The data suggest that, in Australia, the location of the research institution is linked to the contribution of high-impact research. Only four of the Education case studies and two of the Human Society case studies were authored by Australia’s Go8 member universities.

Overall, regional universities are relatively under-represented, constituting less than half of the case studies in Education (n = 7) and Studies in Human Society (n = 4). The under-representation of regional universities in high-for-impact research reflects a need for funding in this area to increase the competitiveness (and impact) of regional universities. Since collaboration is a key factor in high-for-impact research (Figs 1 and 2) and working with national and international research partners often requires funding for collaboration and the establishment of programs for data collection, the question of funding is central to the ability of research institutions to implement high-for-impact research in any area of study.

The geographic distribution of research partners in all studies may reflect the shared problems or issues that research in the fields of Education and Studies in Human Society address concerning public policy and services, health and wellbeing, society, crime, and the economy. Notably, the case studies reflect the concentration of academic knowledge production within Western metropoles. While China, India and some other countries are represented, much of Asia, Africa and Latin America are not represented in these collaborations. There is a bias towards partnerships with researchers and agencies in places where English is spoken as a first language, with minimal engagement with the global south and countries in the Indo-Pacific, despite the region’s strategic geo-political importance to Australia. This may be worth exploring further as a means of aligning future research partnerships with key regional partners. Similarly, most research partners in both areas of study are from UN-Classified developed nations, which is most likely due to the similarity of research foci for Australia as a developed nation, with the most common partner nations also being developed countries (UK, US, New Zealand, Singapore). This under-representation of partnerships with developing or transitional nations limits the scope of research impact in developing countries.

A study by Gonzalez et al. [26] quantified the benefits of partnerships, which included establishing links between collaborative research projects and scientific productivity, as well as increased research impact. Dusdal and Powell [27] further suggest that international research collaborations are associated with higher-quality research than national collaborations and that internationally co-authored papers tend to have increased research impact. It is surprising then, that only around half of the identified high-for-impact research cases involved international research collaborations. One explanation is that such collaboration is time intensive and bureaucratically complex. Those that collaborated internationally tended to have multiple international partners, with five or more partners identified in both fields of study (Fig 2). This suggests that research with five or more international collaborators was more likely to be classified as high-for-impact than studies with fewer international collaborators, particularly for Studies in Human Society (Fig 2). This finding supports research that has found a positive correlation between international collaboration and research quality [28]. The geographical distribution of international collaborators also differs between each study area. Education collaborators were mostly from the UK, US, Singapore, and New Zealand (Figs 1 and 2), perhaps reflecting similar educational models in these countries, and consequently a likely synergy of research aims and impact.

Eighteen and twenty-seven percent of the high-for-impact case studies in Education and Human Societies, respectively, had an Aboriginal and Torres Strait Islander focus or beneficiaries. It should be noted, however, that Aboriginal and Torres Strait Islander-specific impact case studies in these fields of study may have been submitted under *Indigenous Studies* (UoA 45). As such, this review has not captured the number, range, or impact of Aboriginal and Torres Strait Islander-focused research in Education and Studies in Human Society.

### Reported impacts of the case studies

A range of reported impacts were identified in the Education and Human Society case studies across the impact domains of economy, society, culture, national security, public services, health, environment, and quality of life. Reported impacts most frequently concerned the ‘society’ domain. While these findings are useful for understanding the nature of research impact in the case studies examined, a limitation of the current study is that it analysed *self-reported impacts*, as described by the researchers who authored the case studies for the ARC. It did not corroborate these self-reported impacts with evidence of impact (i.e., research outputs) or further analysis of the impacts of the research on participants. The frequency in reporting of societal impacts raises important questions about how researchers themselves define and understand ‘research impact’, particularly in fields such as Education and Human Society, where research impact is not as easily defined or quantifiable as it is in other FoRs, such as Science or Technology.

One of the key problems associated with reporting research impact, as revealed by this study, is a lack of clarity on precisely what constitutes social and cultural impacts. This uncertainty may lead scholars to prepare case studies for impact submission that inadequately present or evidence the contribution that their research might make in the different impact domains identified by the ARC. The ARC defines impact as the “contribution that research makes to the economy, society, culture, national security, public policy or services, health, the environment, or quality of life, beyond contributions to academia” [4] (para7); yet concepts like the economy, society, culture, health, and environment remain highly contested within and among different disciplines and fields of research. The concepts of society and culture encompass very different scholarly understandings and diverse colloquial definitions that make it difficult to determine what might constitute impact in these domains. Within anthropology, for example, culture is holistically viewed as a dynamic set of shared values, ideas and rules through which humans engage with others and organise social relations [24, 25].

Cultural practices are recognised as “hybrid, fluid, and constantly adapting to changes in a global system” [29]. Colloquially, culture denotes outward manifestations of societal practice, such as languages, arts, foods, dress, and customs. These two understandings are interrelated but can be referred to as ‘little-c culture’ (cultural beliefs, practices, and values) and ‘big-C Culture’ (artefacts, products, and customs, such as literature and art), respectively [30]. Anthropological understandings of culture include the big-C areas of product and custom, but do not reduce the concept to outward-facing traditions, products, languages, or heritage. The alignment of culture with language within the Australian Fields of Research (FoR) code (47) denotes big-C concepts like linguistics and literary studies. Small-c cultural concepts are grouped under ‘Human Society’, via sub-disciplines such as anthropology, demography, and gender studies [17]. In contrast, references to the promotion of an “innovation culture and economy” [18] (p19) in the documentation of research priority areas reflect a holistic understanding of *c*ulture.

To identify areas of impact, it is necessary to interrogate these constructs. Firth’s [31] definitions of society and culture can provide guidance here. Firth [31] defines ‘society’ as an “organized set of individuals with a given way of life … [*or*] aggregate of social relations” (p27); whereas culture “is that way of life … [*or*] the content of those relations” (p27). Here, society is defined as a system of social organisation comprised of rules, structures, and institutions. Societal impacts are therefore classifiable as structural or strategic changes within systems of collective organisation, while ‘cultural’ impacts are changes to the shared values, understandings and relations that underpin society; they are distinct, but interdependent and interrelated.

Our examination of the use of the term ‘culture’ in the Education and Human Society case studies, revealed very different understandings of the concept of culture and, thus, cultural impact. For example, in some studies, culture was understood as ‘values’, and the impact was interpreted as a ‘shift’ in values (HCS3). In contrast, other studies appeared to understand culture as expressed in attitudes and behaviours, and cultural impact as being a change in these attitudes and behaviours. In HCS9, for example, the research concerned the “challenge of changing a culture of under-reporting of crimes” [32] in rural areas. Culture, in this case, is understood as human action in the world, or what people do or do not do (i.e., the under-reporting of crime in rural areas). Thus, the contribution to culture, in this case, was an App that enabled human action.

In terms of the problematic distinction between ‘little-c culture’ and ‘big-C culture’, most of the case studies adopted a little-c definition of culture (i.e., practices and values). Two studies took a narrower big-C view of culture (HCS11, ECS16). In HCS11, the research was used by the Western Australian Department of Culture and the Arts as a strategic planning resource and led to new work with industry partners to develop metrics for assessing the impact of the arts on urban life. This was the only study that referred to culture in terms of an ‘arts’ definition. In the Education case studies, reference to culture included the development of culturally-relevant literacy resources in Fiji that supported communities’ heritage languages and cultural identity (ECS2); a pedagogical project that improved ‘teaching cultures’ by addressing student engagement is low-SES contexts (ECS3); and the development of a play-based pedagogical framework that claimed to have achieved major cultural impacts by facilitating early second-language learning in the years before school (ECS16). Here, the impacts can be interpreted as contributing to little-c culture, as they concern people’s everyday behaviour, beliefs, customs, and values.

Many studies did not mention the term ‘culture’ at all, instead claiming contribution to the economy or society. One example is HCS7, which explicitly focused on community engagement initiatives to reduce the negative impacts of fly-in and fly-out mine work on rural communities, while implicitly challenging the cultural construction of masculinity in rural communities and the resources sector. Wider adoption of a holistic anthropological definition of culture which integrates all aspects of culture, as both practice and product, would enhance impact case studies by broadening our understanding of social and cultural impact. Current ARC definitions artificially separate economic, social, and cultural impacts; yet, from a holistic anthropological perspective, these areas must be taken together and understood in relationship to each other. Any research that claims (or seeks) impact in the domain of culture also has impact in the domain of society, and vice versa.

### Evidence cited by researchers to support their claims of research impact

In terms of research outputs, the similarity of the number of outputs in the two different areas of study presents an interesting finding, perhaps reflecting a key cross-discipline indicator for future impact; however, the distributions are noticeably different (Fig 4). This could indicate that evidencing impact in Education might be less reliant on the number of outputs. It could also reflect the difference in the research focus of each area of study. Nevertheless, the reported number of research outputs cited per case study (a median of 10 across the two UoAs) and the number of years between the publication of the outputs and submission of the impact case studies (a median of 12 and 9, respectively, for Education and Human Society) reinforces the view that it takes a collection of research and translation activities over a significant number of years, well beyond the life of a single project, to demonstrate impacts [6]. This highlights a need for researchers to be resourced not only to look back and explore impacts of past research but importantly, to plan for and track impacts into the future [16].

For both short- and long-term research impact to be tracked and assessed, researchers need to be supported to look back on their own research (5, 10 or 20 years thereafter) to identify outputs they believe have achieved impacts; explore the challenges and opportunities involved in demonstrating such impacts; and disseminate results as peer-reviewed research publications [6, 16]. Such research could then inform the design of future projects. Research funders and university authorities need to recognise that if research is to have credible impact, then the nature of that impact must be considered at the design stage. Designing impactful research will require a system of seed funding prior to the submission of grant applications, and research design must be appropriately resourced to ensure equity across research groups and institutions [6, 16, 33]. The representation of non-Group of 8 institutions in the high-for-impact case studies suggests that further investment of seed funds in these institutions is warranted.

Broad categories for the evidence supporting impact claims in each study were identified and comparisons between the types of evidence used showed key similarities and differences across the two areas. The most common evidence of impact cited in both Education and Human Society combined is the number of surveys/feedback/testimonials and interviews, and the number of users affected (Table 3). Key differences between the evidence types cited by each study area are the number of users affected (more in Education), citations (more in Education), government-supported resources/implemented models (more in Human Society) and development of new initiatives/programs (more in Education). These numbers likely reflect the difference in the research focus of each field of research. However, the types of evidence most associated with high-for-impact research, both overall and for each field, provide valuable insight for researchers as to the types of outputs that support impact claims.

In Education, much of the impact was shown through research that changed policies. This was described in terms of specific policy development (ECS9), the application of new frameworks (ECS15), frameworks impacting Government policy (ECS17), integration of information communication technologies in the national curriculum (ECS16), answering questions for policymakers and developing a needs-based funding model (ECS1), or changes in funding models in five Australian states and territories (ECS2). For the Human Society case studies, examples of policy impact included a new regulatory framework (HCS2), a UN Global Compact Cities Programme to shift approaches to sustainability, and a framework that assigns cultural and political issues equal weight with economic and ecological issues (HCS3). While there was variation in whether these were termed policy, framework or curriculum changes, the key point here is that the case studies reported an uptake of research that led to societal change.

Overall, the analysis of Education and Human Society case studies reveal key features and attributes of impactful research from the ARC point of view. These include the institutional location and support for teams producing the research, the underpinning partnerships and collaborations, funding sources, outputs produced in the form of publications and reports, the time it takes from doing research to achieving impacts, the range of policy and practice impacts or changes resulting from research, and the strengths and limitations of the evidence supporting impact claims. Researchers can use this information to inform more impactful research design which meets what the ARC framework values. What is not clear, though, is how impactful the research was, or how closely the identified impact is related to what was needed by the identified research users. Thus, the capacity of the ARC framework alone to help researchers improve research value and reduce waste is limited, not least because of a lack of explicit and transparent priority-setting in the ARC assessment framework.

An alternative approach would be integrating and seeking evidence against the Lowitja Institute research-for-impact framework, which, among other attributes, takes a radically different approach to research priority-setting based on explicit evidence gap analysis [1]. Research users (whether individuals, civil society, businesses, services or governments) require information to make informed decisions. Crucially, the Lowitja Institute tool asks whether existing knowledge is enough to support such decisions; if yes, how to use it and with what consequences, and if not, what additional information is required, and how to generate and use it. Research impact, from this point of view, is the extent to which research (whether existing and/or new) supports or facilitates better decisions regarding alternative choices of actions facing research users in the first place, and the resulting benefits versus costs, intended or unintended. Based on the Lowitja approach, a large proportion of the research currently being done ought to be simply translating existing knowledge to support competing decision choices, rather than doing more ‘research’ as we know it today [1].

In the face of unprecedented global challenges, it is understandable that governments increasingly endorse research directly impacting policy and practice. Despite corporate influence on higher education, the research impact agenda warrants critical examination, rather than outright dismissal as neoliberal [34]. However, as a social process, the research-practice nexus is far more nuanced than the ARC impact assessment framework implies. Akkerman and colleagues introduce an alternative through ontological synchronization, emphasizing continuous dialogical attunement to societal dynamics [35]. This aligns with the Lowitja research impact tool [1] reframing impact as a "wicked" rather than a ‘technical’ problem, requiring participatory, trust-based approaches. The establishment of collaborative spaces and deep learning processes is crucial for addressing complex issues. Embracing Indigenous research principles, the Lowitja Institute’s approach to impact prioritizes cultural congruence and Indigenous leadership, challenging traditional researcher-led processes. For instance, instead of directly funding researchers, Lowitja now supports research user organizations, empowering them through direct funding to invite researchers as partners. Additionally, before initiating conventional research, teams must assess the adequacy of existing knowledge (experiential and research) for the identified research need, emphasizing transparency and minimizing research waste [36].

## Limitations

This review focused on two fields of research, Education and Studies in Human Society; therefore, the implications drawn from this study are focused on these specific fields of practice. In addition, the study sought to assess reported impacts in the form of case study submissions, rather than seeking to independently measure or verify reported impacts. While the number of cases in each field is relatively small, general insights can be drawn to help researchers understand the evidence needed to demonstrate impact, and to generate reflection and discussion about impact and funding models for future research. In addition, the robust methodological approach adopted in the current study, combined with anthropological understandings of culture, has produced a review that is not only trustworthy, but offers useful insights and implications beyond the fields of Education and Studies of Human Society.

## Conclusion

The ARC *Engagement and Impact Assessment* framework [4] incentivises research that brings about tangible impacts. While there is a risk that focusing on impact as a core funding metric may privilege applied research with quantifiable outcomes over harder-to-measure social or cultural changes, the ARC impact framework has brought several benefits for education and social science research. For example, it encourages researchers to develop collaborations that will positively impact communities, and it presents a valuable opportunity to drive societal change through high-for-impact research that is values-driven (e.g., research that promotes social justice and equity). The framework has also generated a set of case studies for analysis that are valuable for informing future research. By exploring high-for-impact studies submitted for two fields of research, Education and Studies in Human Society, the current study may support the identification of impacts that are underrepresented in past research, and that may be used to inform research design and future research agendas.

Interrogating the publicly available research impact case studies submitted in previous Engagement and Impact rounds is a useful means of developing impact literacy among researchers. Improved impact literacy among social researchers would be beneficial, especially regarding the interpretation, recognition, and evidencing of impact; however, the impact domains identified by the ARC remain relatively ambiguous and open to interpretation, which leaves scope for researchers to evidence the impact of their research in ways that transcend the limited categories of evidence presented in past case studies. We have argued, for example, that a more holistic understanding of the impact domain of ‘culture’ and the complex interconnections between culture and other domains identified by the ARC, would enhance recognition of potential impacts. Improved understandings of what constitutes impact will lead to better case study development and evidencing of impact claims. Wider adoption of the holistic anthropological definition of culture, which integrates values, practices, and products, would enhance impact case studies by expanding their focus to include the broader cultural changes which underpin sustained social change. Ultimately, improving the value of research for society will require a reconceptualisation of research impact and its role in allocating funding. Assessments of research impact should be orientated towards exploring the benefits and costs arising from better or smarter decisions and actions, without neglecting to document the intended and unintended, positive, or negative impacts of research over time, and the Lowitja Institute’s research-for-impact framework provides a positive starting point for such analysis.
